# Correction: Laser cleavable probes for *in situ* multiplexed glycan detection by single cell mass spectrometry†

**DOI:** 10.1039/c9sc90270h

**Published:** 2020-01-09

**Authors:** Jing Han, Xi Huang, Huihui Liu, Jiyun Wang, Caiqiao Xiong, Zongxiu Nie

**Affiliations:** Beijing National Laboratory for Molecular Sciences, Key Laboratory of Analytical Chemistry for Living Biosystems, Institute of Chemistry, Chinese Academy of Sciences Beijing 100190 China znie@iccas.ac.cn; University of the Chinese Academy of Sciences Beijing 100049 China; National Center for Mass Spectrometry in Beijing Beijing 100049 China

## Abstract

Correction for ‘Laser cleavable probes for *in situ* multiplexed glycan detection by single cell mass spectrometry’ by Jing Han *et al.*, *Chem. Sci.*, 2019, **10**, 10958–10962.

The authors regret that there is some ambiguity over the origin of the probe molecule and would like to clarify that in the manuscript they presented a multiplexed and sensitive glycan detection approach based on laser cleavable probes.

In the article a multiplexed and sensitive glycan detection approach based on laser cleavable probes is developed. In this method, four kinds of glycans at the surface of single cell and tissue were recognized by their specific lectins, and sensitive mass spectrometry detection was achieved by combining the lectins with laser cleavable probes with different mass tags.

Regarding the synthesis of the laser cleavable probes, the four laser cleavable probes **1–4** were previously synthesized by Moon *et al.*^1^ (reference 31 in the original article) and details of the syntheses are provided in the ESI (Scheme S1 and Fig. S1–S8†). These probes were chosen as the mass tags to detect glycans for the first time.

The Royal Society of Chemistry apologises for these errors and any consequent inconvenience to authors and readers.

## Supplementary Material

SC-011-C9SC90270H-s001
