# Supplementary material for: Correction: Laser cleavable probes for in situ multiplexed glycan detection by single cell mass spectrometry
Source: Chem Sci. 2020 Jan 9;11(4):1176. doi: 10.1039/c9sc90270h (PMC8146070; doi:10.1039/c9sc90270h)
Supplement: SC-011-C9SC90270H-s001 [file SC-011-C9SC90270H-s001.pdf]

Electronic Supplementary Material (ESI) for Chemical Science.
This journal is © The Royal Society of Chemistry 2020

Supporting Information for:

Laser Cleavable Probe for *in-Situ* Multiplexed Glycan Detection by Single Cell Mass Spectrometry

Jing Han,[a,b] Xi Huang,[a] Huihui Liu,[a] Jiyun Wang, [a] Caiqiao Xiong* [a] and Zongxiu Nie*[a,b,c]

[a] Beijing National Laboratory for Molecular Sciences, Key Laboratory of Analytical Chemistry for Living Biosystems, Institute of Chemistry, Chinese Academy of Sciences, Beijing 100190, China. E-mail: znie@iccas.ac.cn

[b] University of the Chinese Academy of Sciences, Beijing 100049, China.

[c] National Center for Mass Spectrometry in Beijing, Beijing 100190, China

Table of contents

1. Experimental……………………………………………………………........................S3
2. Figure S1………………………………………………………………….....................S10
3. Figure S2……………………………………………………………...…......................S10
4. Figure S3..……………………….........…………………………………………….S11
5. Figure S4…...…………………………………………….……….………………S11
6. Figure S5……....………………………….………………………..……..................S12
7. Figure S6………...…………………….……………………….…………………...S12
8. Figure S7…………..……………………….…………………………………….S13
9. Figure S8……………………………………………………..……...............…...S13
10. Figure S9……………...…………………………….………………………………...S14
11. Figure S10……………..………………………….………………….……………S14
12. Figure S11……………………..…………………………………….………….S15
13. Figure S12…………………...……………………………………….………….S15
14. Figure S13……………………...……...……………………………….………….S16
15. Figure S14……………………...……………………………………….………….S16
16. Figure S15………………………...…………………………………….………….S17
17. Figure S16……………………..…………………………………….………….S17
18. Figure S17……………………...………………………………….………….S17
19. Figure S18……………………...……………...……………….………….S18
20. Figure S19…...……………………………………......………………………...S18
21. Figure S20…..S19
22. Figure S21…..S20
23. Figure S22…..S21
24. Figure S23…..S21
25. Figure S24…..S21
26. Figure S25…..S22
27. Figure S26…..S22
28. Table S1…...S23
29. Table S2..……..S23
30. Reference…...S23

Experimental

Reagents and apparatus:

Thiochro-man-4one, phenyl hydrazine, trimethylsilyl chloride, NaH, iodomethane, trifluoroacetic anhydride (TFAA), 1-bromopropane, 1-iodobutane, 3-chloroperbenzoic acid, iodoethane, sodium thiosulfate and 3-mercaptopropionic acid were obtained from Beijing InnoChem Science & Technology Co., Ltd. N-hydroxysuccinimide (NHS), *N*-(3-Dimethylaminopropyl)-*N'*-ethylcarbodiimide hydrochloride (EDCI), hydrochloride, DMSO-d_6, bovine serum albumin (BSA), *N,N*-dimethylformamide (DMF), concanavalin A (ConA), ricinus communis agglutinin (RCA_{120}), wheat germ agglutinin (WGA) and elderberry (SNA) were obtained from Sigma-Aldrich. NaCl, Na_2SO_4, $NaHCO_3$, Na_2CO_3, ethyl acetate (EtOAc), hexane, ethanol (EtOH), CH_3CN, and diethyl ether (Et_2O) were obtained from Beijing Chemicals, Ltd. MCF-7 (breast cancer) cell lines were purchased from the Cell Resource Center, Shanghai Institute for Biological Sciences (Chinese Academy of Sciences, Shanghai, China). MCF-7R (Doxorubicin-resistant MCF-7 subline) cell lines were purchased from Shanghai Aiyan Biological Technology Co. Ltd. (Shanghai, China). RPMI-1640 medium, 10% fetal bovine serum and 1% penicillin/streptomycin were purchased from Thermo Fisher Scientific Co., Ltd. Ultrapure water (over 18 MΩ·cm) from a Milli-Q reference system (Millipore) was used throughout. The stock solution (1 mM) of probes **1-4** were prepared in DMF.

Matrix-assisted laser desorption/ionization mass spectrometry (MALDI MS) was performed on a Bruker Microflex time-of-flight mass spectrometer (Bruker Daltonics, Bremen, Germany) equipped with a 355 nm and 2 kHz solid state Nd:YAG Smart Beam laser. The mass spectrum was summed up by 400 shots at a laser repetition rate of 1000 Hz and analyzed by flexAnalysis (Bruker Daltonics, Germany). ^1H-NMR and ^{13}C-NMR spectra were measured with a Bruker DMX-400 spectrometer. Diffusion-ordered NMR spectroscopy was recorded with a Bruker DMX-600 spectrometer. Fluorescence imaging was conducted on an FV 1000-IX81 confocal laser scanning microscope (Olympus, Japan). Absorption spectra were made by microplate reader (Molecular Devices SpectraMax i3). The fluorescence intensity of cells was determined by a Becton Dickinson FACScalibur flow cytometer (Becton Dickinson, USA). Circular dichroism was measured by J-815 Circular dichroism spectrometer (JASCO, Japan).

Syntheses of probes

Probes **1-4** were prepared according to the reported literature. [1]

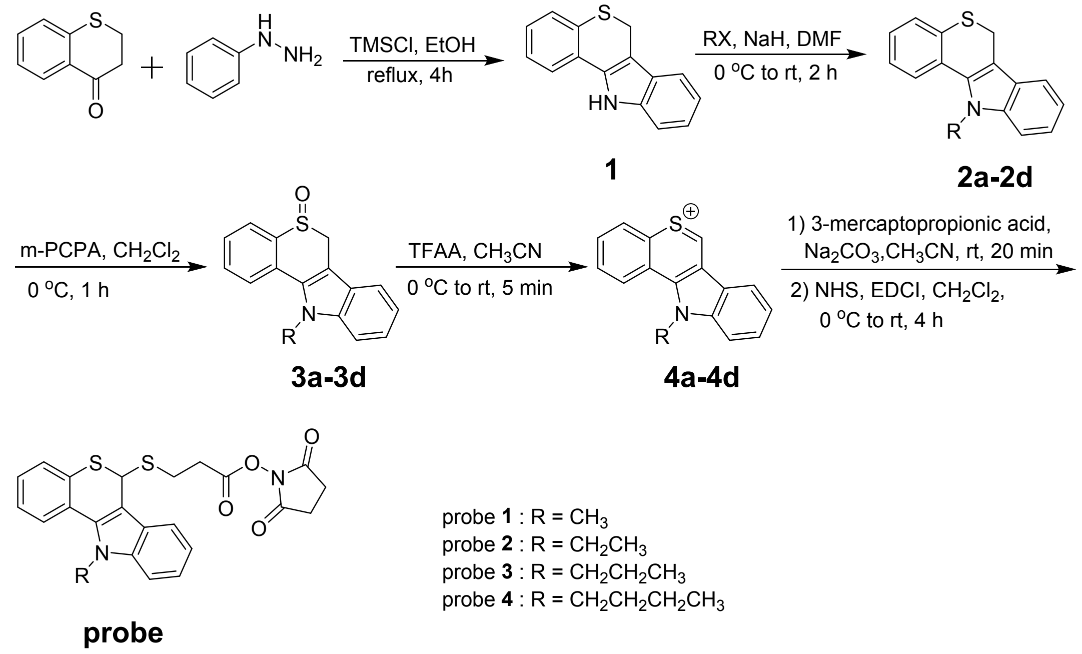

Scheme S1. The illustration of synthetic route of probes **1-4**.

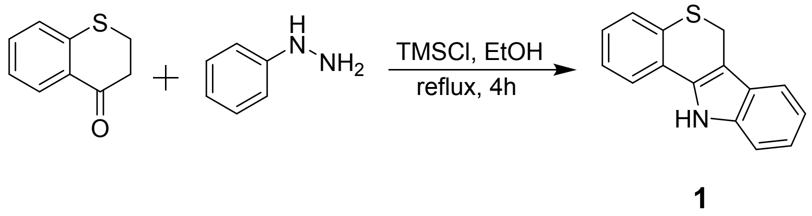

1: Thiochro-man-4-one (0.822 g, 5 mmol) was placed round bottom flask with EtOH (10 mL), phenyl hydrazine (0.541 g, 5 mmol) and trimethylsilyl chloride (0.543 g, 5 mmol) were added to the solution, respectively. The reaction mixture was heated to reflux for 4 h. After completing it, the solution was basified with saturated $NaHCO_3$ solution and diluted with CH_2Cl_2 (10 mL). The organic layer was separated and the aqueous solution was washed with 10 mL CH_2Cl_2 twice. The totally collected organic solvent was dried over anhydrous Na_2SO_4. The solvent was removed by evaporation under reduced pressure, and the residue was subjected to silica gel chromatography with eluent (EtOAc: hexane, v/v, 1:20 to 1:5), affording **1** as a white solid (1.067 g, 90%).

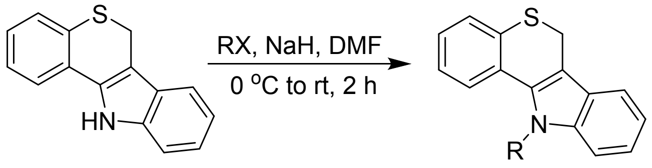

2a : R = CH_3
2b : R = CH_2CH_3
2c : R = $CH_2CH_2CH_3$
2d : R = $CH_2CH_2CH_2CH_3$

1 **2a-2d**

2a-2d: The mixture of (**1**, 1.42 g, 6 mmol) and NaH (0.530 g, 12 mmol) in anhydrous DMF (15 mL) was stirred for 30 min at 0 ℃ under N_2. Then, alkyl halide (12 mmol) was added to the solution, and the reaction mixture was stirred at RT for 2 h. Subsequently, H_2O (10 mL) and CH_2Cl_2 (40 mL) were sequentially added to the solution. The organic layer was collected and dried over anhydrous Na_2SO_4. The solvent was removed by evaporation, and the residue was subjected to silica gel chromatography eluted with EtOAc: hexane (v/v, 1:20), obtaining **2a-2d** as a white solid, which was used without purification.

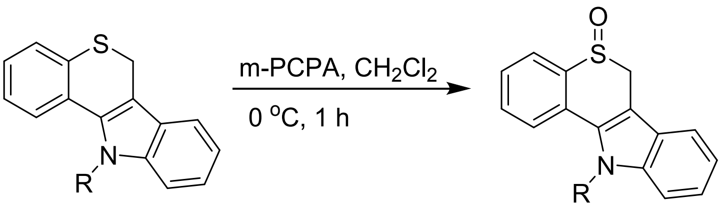

3a : R = CH_3
3b : R = CH_2CH_3
3c : R = $CH_2CH_2CH_3$
3d : R = $CH_2CH_2CH_2CH_3$

2a-2d **3a-3d**

3a-3d: (**2a-2d**, 1.0 equiv) was dissolved in anhydrous CH_2Cl_2, and 3-chloroperbenzoic acid (69%, 1.1 equiv) was added to the solution at 0 ℃. The mixture was stirred for 1h, and then, 20% sodium thiosulfate aqueous solution was added to the solution to quench the reaction. The organic layer was collected and dried over anhydrous Na_2SO_4. The solvent was removed by evaporation, and the residue was purified using silica gel chromatography with EtOAc: hexane (v/v, 1:1) as eluent, was obtained **3a-3d** as a yellow solid, which was used without purification.

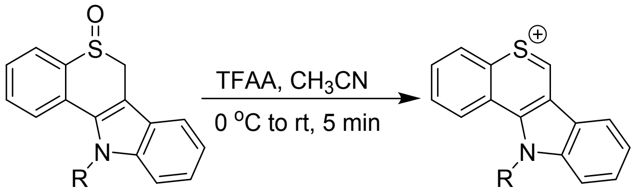

4a : R = CH_3
4b : R = CH_2CH_3
4c : R = $CH_2CH_2CH_3$
4d : R = $CH_2CH_2CH_2CH_3$

3a-3d **4a-4d**

4a-4d: (**3a-3d**, 1.0 equiv) and TFAA (3.0 equiv) were dissolved in CH_3CN at 0 ℃ under N_2. The mixture was stirred for 5 min, and then the deep yellow solution was concentrated by evaporation, and the crude product was precipitated in Et_2O at 0 ℃.

The resulting yellow solid was filtered, washed with cold anhydrous Et_2O, and dried under reduced pressure to give the thionium salt **4a-4d** as a deep yellow solid, which was used without further purification.

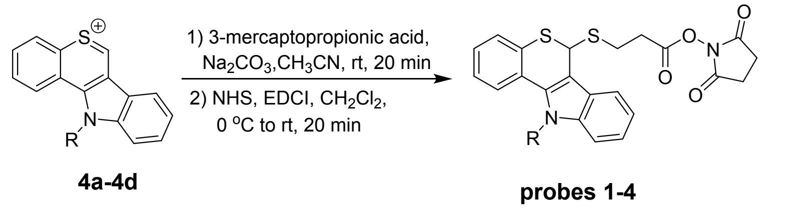

probe **1** : R = CH_3
probe **2** : R = CH_2CH_3
probe **3** : R = $CH_2CH_2CH_3$
probe **4** : R = $CH_2CH_2CH_2CH_3$

4a-4d **probes 1-4**

Thionium salt (**4a-4d**, 1.0 equiv), 3-mercaptopropionic acid (1.0 equiv), and Na_2CO_3 (1.0 equiv) was dissolved in CH_3CN, and then the mixture was stirred at RT until solution turns to colorless and diluted with CH_2Cl_2. H_2O was added to the mixture until all the solid was dissolved. Subsequently, the organic layer was collected and washed with saturated NH_4Cl aqueous solution. The organic solution was dried over anhydrous Na_2SO_4, and the solvent was evaporated under reduced pressure. The resulting solid was used without further purification.

A mixture of the crude carboxylic acid and N-hydroxysuccinimide (NHS) (1.1 equiv) was dissolved in anhydrous CH_2Cl_2, followed by the addition of EDCI (3.0 equiv) in CH_2Cl_2 via cannula at 0 ℃ under N_2. The resulting solution was stirred at RT for 4 h. After that, the solution was diluted with CH_2Cl_2 and washed twice with H_2O. The collected organic solution was dried over anhydrous Na_2SO_4. The solvent was evaporated under reduced pressure and the residue was purified using silica gel chromatography with EtOAc: hexane (v/v, 1:1) as eluent, obtaining **probes 1-4** as a white solid.

Probe **1:** Yield 51%. The ^{1}H-NMR and ^{13}C-NMR spectra of probe **1** are shown below in Figures S1 and S2, respectively. ^{1}H-NMR (400 MHz, 298 K, DMSO-d_6): δ 7.98 (d, J=7.7 Hz,1H), 7.72 (d, J=7.9 Hz, 1H), 7.60 (d, J=7.8 Hz, 1H), 7.57 (d, J=8.3 Hz, 1H), 7.43 (dd, J=7.7, 7.6 Hz, 1H), 7.35 (dd, J=6.9, 7.5 Hz, 1H), 7.29 (dd, J=7.2, 7.5 Hz, 1H), 7.17 (dd, J=7.6, 7.2 Hz, 1H), 6.23 (s, 1H), 4.00 (s, 3H), 3.24-3.09 (m, 2H), 3.03-2.96 (m, 1H), 2.89-2.75 (m, 5H). ^{13}C-NMR (100 MHz, 298 K, DMSO-d_6): δ 170.0, 167.7, 138.1, 134.5, 130.3, 129.5, 127.4, 126.6, 126.5, 124.9, 122.8, 122.6, 120.0, 118.5, 110.6, 110.2, 42.8, 32.8, 31.3, 25.6, 25.3.

Probe **2**: Yield 45%. The ^{1}H-NMR and ^{13}C-NMR spectra of probe **2** are shown below in Figures S3 and S4, respectively. ^{1}H-NMR (400 MHz, 298K, DMSO-d_6): δ 7.82 (d, J=7.7 Hz,1H), 7.72 (d, J=7.9 Hz, 1H), 7.61 (t, J=7.5 Hz, 2H), 7.46 (dd, J=6.7,

7.6 Hz, 1H), 7.35 (dd, J=7.6, 7.3 Hz, 1H), 7.29 (dd, J=7.8, 7.5 Hz, 1H), 7.18 (dd, J=7.6, 7.2 Hz, 1H), 6.22 (s, 1H), 4.47 (q, J=6.8 Hz, 2H), 3.23-3.08 (m, 2H), 3.05-2.96 (m, 1H), 2.82-2.76 (m, 5H), 1.42 (t, J=7.1 Hz, 3H). ^{13}C-NMR (100 MHz, 298 K, DMSO-d_6): δ 170.6, 168.3, 138.0, 134.4, 131.1, 130.3, 128.1, 127.4, 127.2, 124.9, 123.5, 123.5, 120.8, 119.3, 117.7, 110.9, 43.3, 35.4, 31.9, 26.2, 25.9, 15.9.

Probe **3**: Yield 59%. The ^{1}H-NMR and ^{13}C-NMR spectra of probe **3** are shown below in Figures S5 and S6, respectively. ^{1}H-NMR (400 MHz, 298 K, DMSO-d_6): δ 7.84 (d, J=7.8 Hz, 1H), 7.72 (d, J=7.8 Hz, 1H), 7.60 (t, J=7.8 Hz, 2H), 7.45 (dd, J=6.9, 7.4 Hz, 1H), 7.34 (dd, J=7.5, 7.2 Hz, 1H), 7.28 (dd, J=7.2, 7.5 Hz, 1H), 7.17 (dd, J=7.6, 7.2 Hz, 1H), 6.21 (s, 1H), 4.46-4.31 (m, 2H), 3.23-3.08 (m, 2H), 3.03-2.96 (m, 1H), 2.82-2.75 (m, 5H), 1.85-1.67 (m, 2H), 0.84 (t, J=7.3 Hz, 3H). ^{13}C-NMR (100 MHz, 298 K, DMSO-d_6): δ 170.8, 168.3, 138.5, 134.6, 131.0, 130.3, 128.0, 127.4, 127.3, 124.7, 123.5, 123.4, 120.7, 119.3, 112.0, 111.1, 46.7, 43.3, 32.0, 26.3, 26.0, 23.6, 11.4.

Probe **4**: Yield 50%. The ^{1}H-NMR and ^{13}C-NMR spectra of probe **4** are shown below in Figures S7 and S8, respectively. ^{1}H-NMR (400 MHz, 298 K, DMSO-d_6): δ 7.85 (d, J=7.8 Hz,1H), 7.73 (d, J=7.7 Hz, 1H), 7.60 (t, J= 8.1 Hz, 2H), 7.44 (dd, J=7.3, 7.0 Hz, 1H), 7.33 (dd, J=7.5, 7.5 Hz, 1H), 7.28 (dd, J=7.3, 7.9 Hz, 1H), 7.17 (dd, J=7.8, 7.2 Hz, 1H), 6.21 (s, 1H), 4.48-4.34 (m, 2H), 3.24-3.09 (m, 2H), 3.04-2.97 (m, 1H), 2.90-2.76 (m, 5H), 1.81-1.62 (m, 2H), 1.30-1.16 (m, 2H), 0.87 (t, J=7.3 Hz, 3H). ^{13}C-NMR (100 MHz, 298 K, DMSO-d_6): δ 170.6, 168.3, 138.4, 134.7, 131.0, 130.3, 128.0, 127.4, 127.2, 124.8, 123.5, 120.8, 119.3, 112.0, 111.1, 45.0, 43.4, 32.3, 32.0, 26.3, 26.0, 19.8, 14.0.

Lectin-probes conjugation

Lectins including ConA, RCA_{120}, WGA and SNA were first dissolved in PBS (pH 7.4) at a concentration of 0.2 mg/mL. 50 μL, 20 μmol/mL probes **1-4** was added to the lectin solution respectively, and incubated at room temperature for 3 h in dark. Using a 10 KD Ultra Centrifugal Filter (Merck Millipore, Germany), excess probes were removed and solution was buffer exchanged to PBS (pH 7.4) to make the ConA-probe**1**, RCA_{120}-probe**2**, WGA-probe**3** and SNA-probe**4** conjugate, respectively. Protein concentration was determined using Bradford protein assay kit (Solarbio, Beijing).

Cell culture

MCF-7 cells and MCF-7R cells were cultured in RPMI 1640 medium supplied with 10% fetal bovine serum and 1% penicillin/streptomycin at 37 ºC in a humidified atmosphere of 5% CO_2.

Confocal Microscopy Imaging

MCF-7 cells and MCF-7R cells were seeded at desired concentrations in covered glass-bottomed cell confocal dishes. After cultured for at least 24 h, the cells were washed three times by PBS and stained by FITC-labeled ConA-probe**1** for 30 min. After removing excess FITC-labeled ConA-probe**1** by washing with PBS, cells were imaged with a laser scanning confocal microscope.

Monosaccharide inhibition assay

1 μg/mL FITC-labeled ConA-probe**1** was pre-incubated with 1 mg/mL free monosaccharides (α-methyl-mannoside, α-methyl-glucoside and D-galactose) respectively at 37 °C for 1 h. After the removal of excess monosaccharides by 10KD Ultra Centrifugal Filter, the conjugates were incubated with MCF-7 cells at 37 °C for 30min. The cell suspension was centrifuged at 1000 rpm for 5 min, washed twice, resuspended in PBS buffer and filtered by 400-mesh sieve. The fluorescence intensity of cells was determined by a Becton Dickinson FACScalibur flow cytometer. For each flow cytometric test sample, 10,000 events were acquired, and the mean fluorescence intensity was used for analysis.

Tunicamycin treatment

MCF-7 cells were cultured in cell culture medium in the absence and presence of tunicamycin of different concentration (20 μg/mL, 50 μg/mL, 100 μg/mL, 200 μg/mL) for 24 h.

LDI-MS analysis: cells were trypsinized and incubated with ConA-probe**1** for 40 min. After washing three times with PBS buffer, cells were directly analyzed by LDI-MS.

Flow cytometry analysis: cells were trypsinized and incubated with FITC-labeled ConA-probe**1** at 37 °C for 30min. The cell suspension was centrifuged at 1000 rpm for 5 min, washed twice, resuspended in PBS buffer and filtered by 400-mesh sieve. The fluorescence intensity of cells was determined by a Becton Dickinson FACS calibur flow cytometer. For each flow cytometric test sample, 10,000 events were acquired, and the mean fluorescence intensity was used for analysis.

Viability assay: Cells were seeded in 96-well plate and cultured for 24 h. After washing with PBS, cells were incubated with different concentration of tunicamycin (0, 1, 10, 50, 75, 100 μg/mL) for 24 h. 10 μL CCK-8 (Cell Counting Kit-8) reagent was added into each well with 100 μL cell culture medium inside. Absorbance at 450 nm was measured by a microplate reader after 1 h incubation.

LDI-MS analysis

MCF-7 cells and MCF-7R cells were seeded at desired concentrations and trypsinized. Lectin-probe was added and incubated with cells at 37 °C for 30min. After washing three times with PBS buffer, cells were directly analyzed by LDI-MS.

Single cell analysis

MCF-7 cells and MCF-7R cells were seeded on the indium tin oxide (ITO)-coated glass slides at desired concentrations for 24 h. To reduce cell-to-cell contamination during MS analysis, cells were ≥20 μm (two-fold greater than the diameter of the laser probe) away from other cells. Lectin-probe was added and incubated with cells at 37 °C for 30min. After washing three times with PBS buffer, cells were imaged by a laser scanning confocal microscope, then analyzed by LDI-MS. The "small" (~10 μm footprint) laser setting was used and 400 laser shots were accumulated at 1000 Hz and 20% laser energy for each cell. After MS analysis, the optical imaging was performed to confirm single cell was analyzed by one laser shot.

LDI imaging mass spectrometry

Human breast tissue was provided by the Peking University Third Hospital. Fresh-frozen tissue was cut at 10 μm using a Leica CM1950 cryostat (Leica Microsystems GmbH, Wetzlar, Germany) at -20 ^{o}C and thaw mounted onto indium tin oxide (ITO) coated glass slide. The certain amount of lectin-probe conjugates was added to the surface of tissues and incubated at 37 ℃ for an hour. Then, tissues were gently washed by PBS buffer, and the glass slides were placed into a vacuum desiccator for approximately 30 minutes before LDI-MS analysis.

Supplementary Figures:

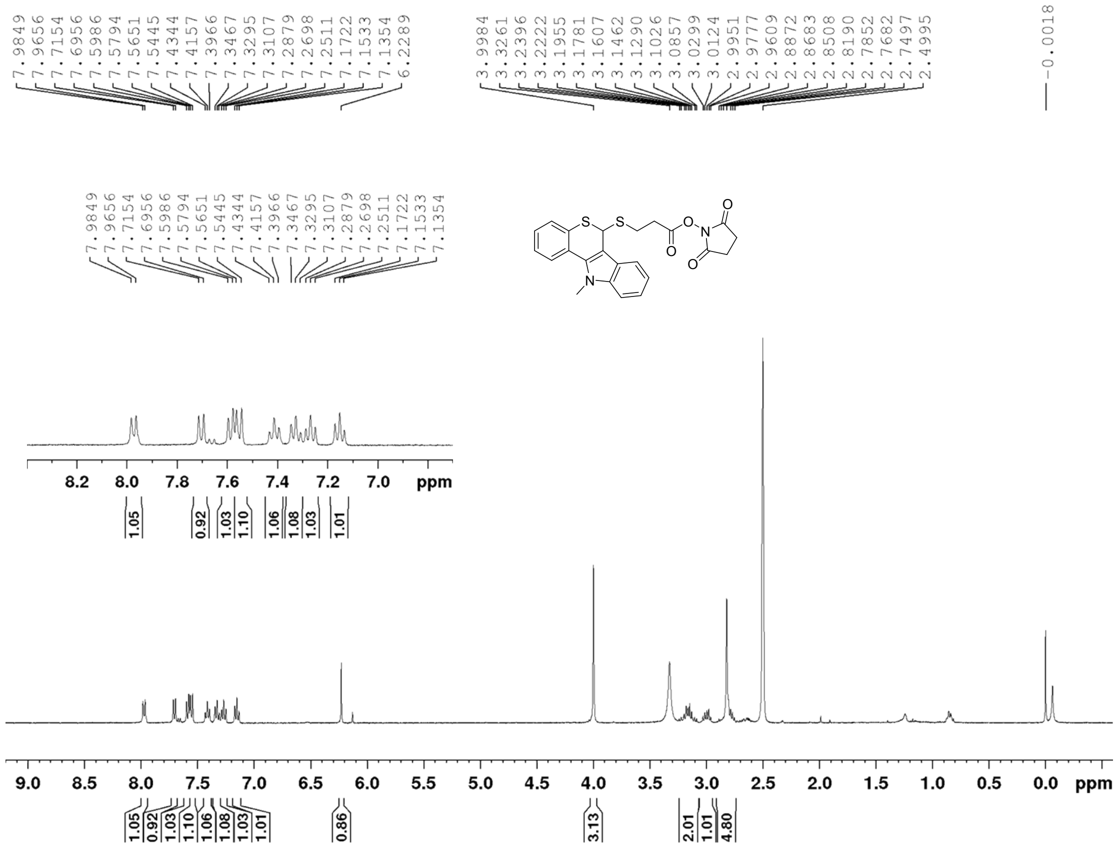

Figure S1. ^{1}H-NMR spectrum of probe **1** (400 MHz, 298 K, DMSO-d_6).

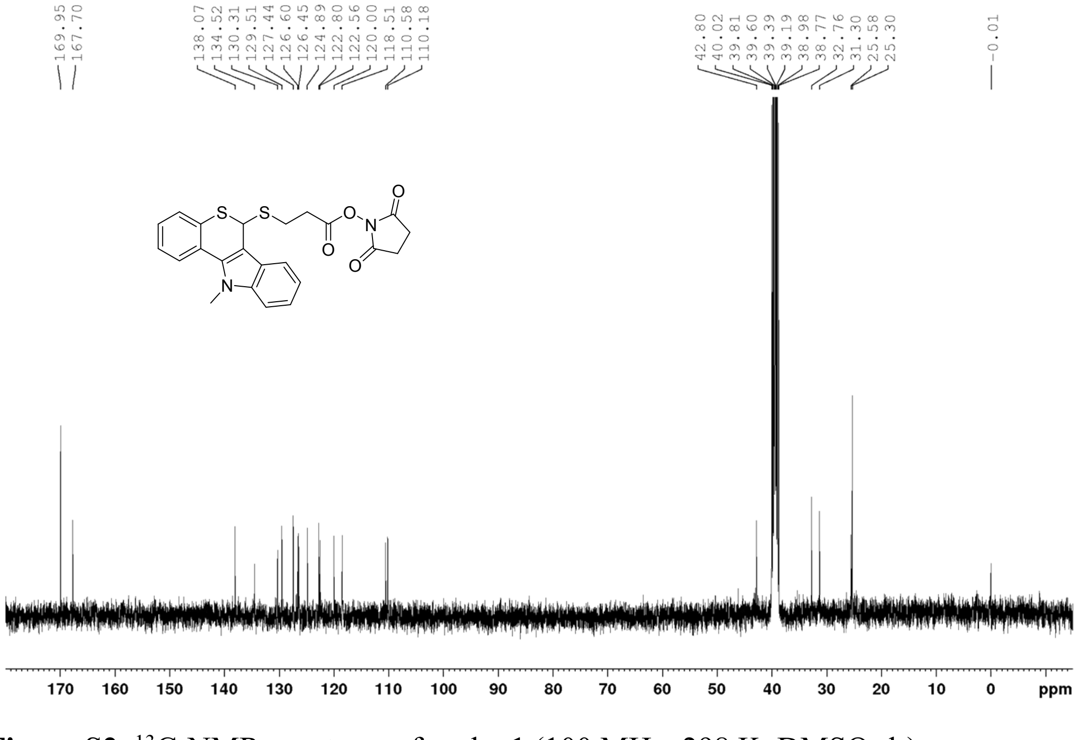

Figure S2. ^{13}C-NMR spectrum of probe **1** (100 MHz, 298 K, DMSO-d_6).

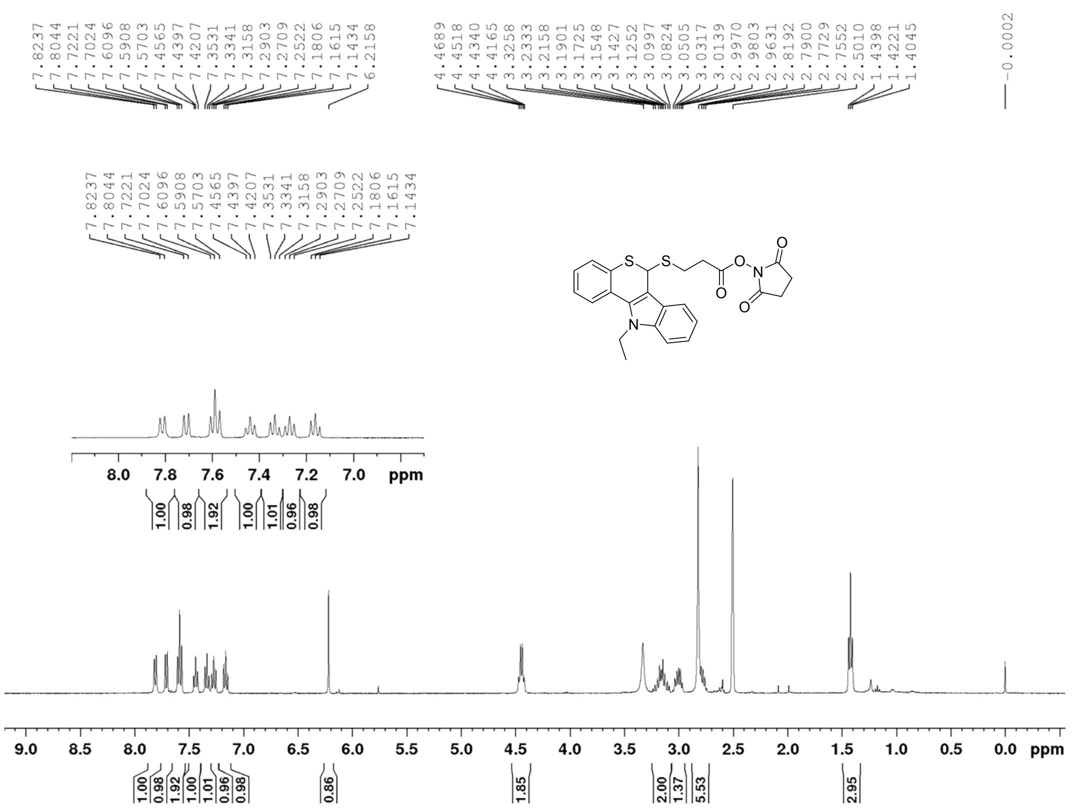

Figure S3. ^{1}H-NMR spectrum of probe **2** (400 MHz, 298 K, DMSO-d_6).

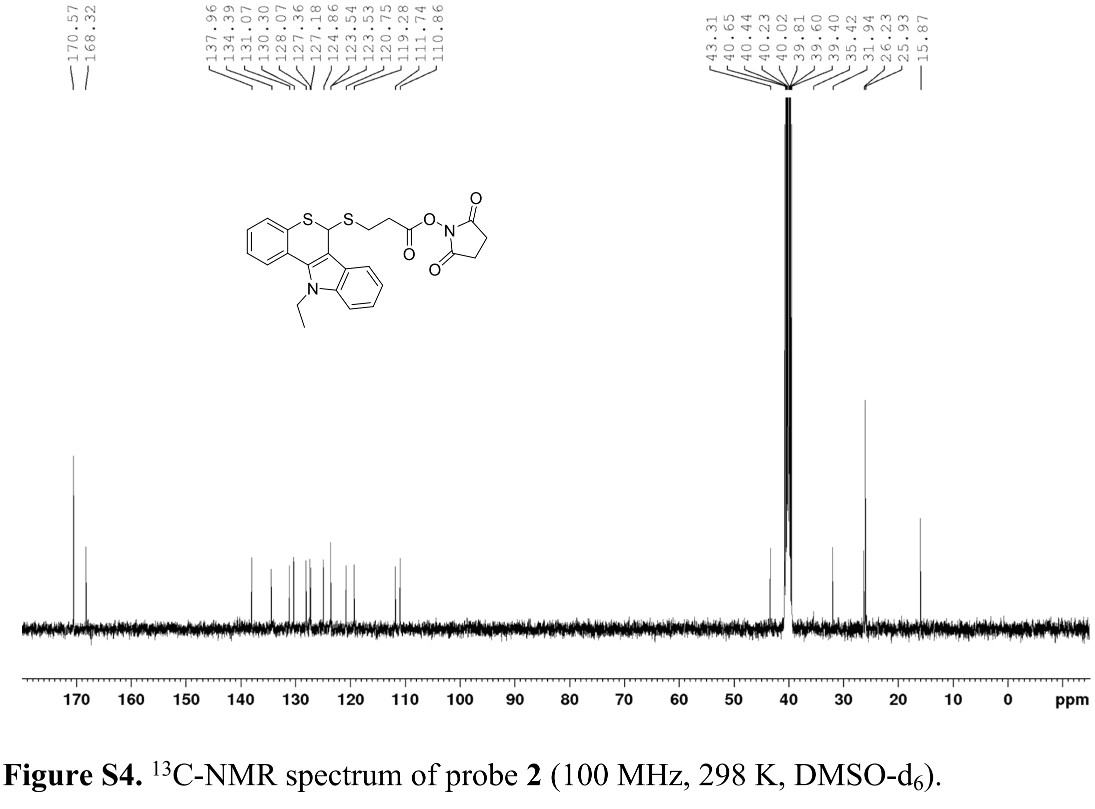

Figure S4. ^{13}C-NMR spectrum of probe **2** (100 MHz, 298 K, DMSO-d_6).

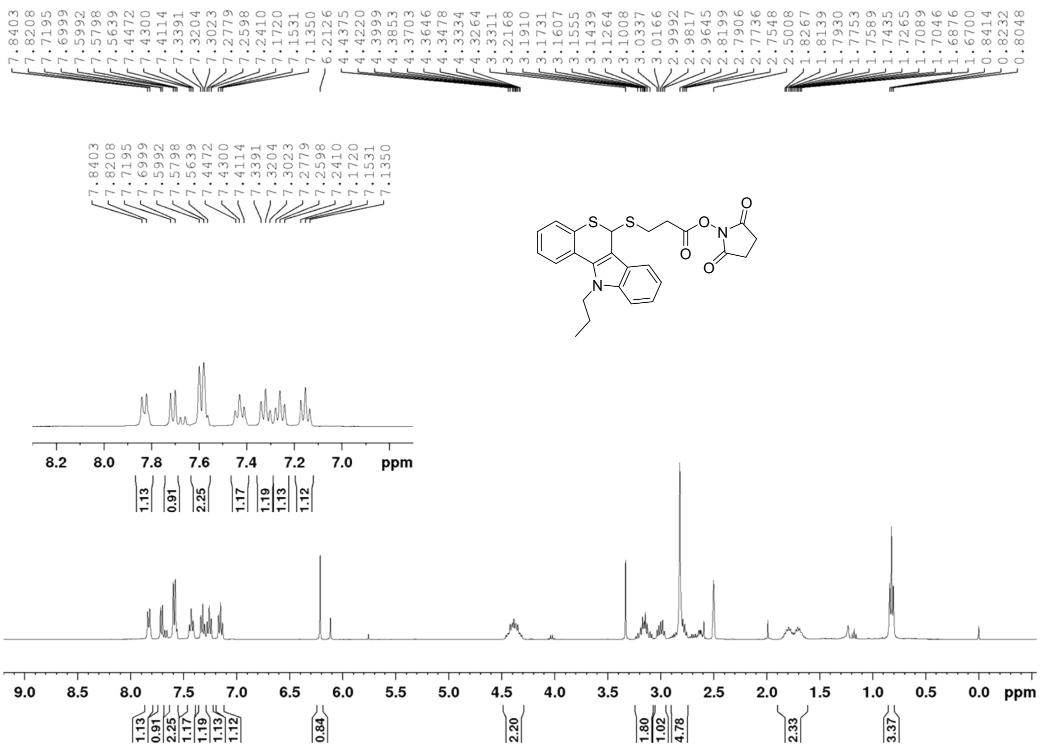

Figure S5. ^{1}H-NMR spectrum of probe **3** (400 MHz, 298 K, DMSO-d_6).

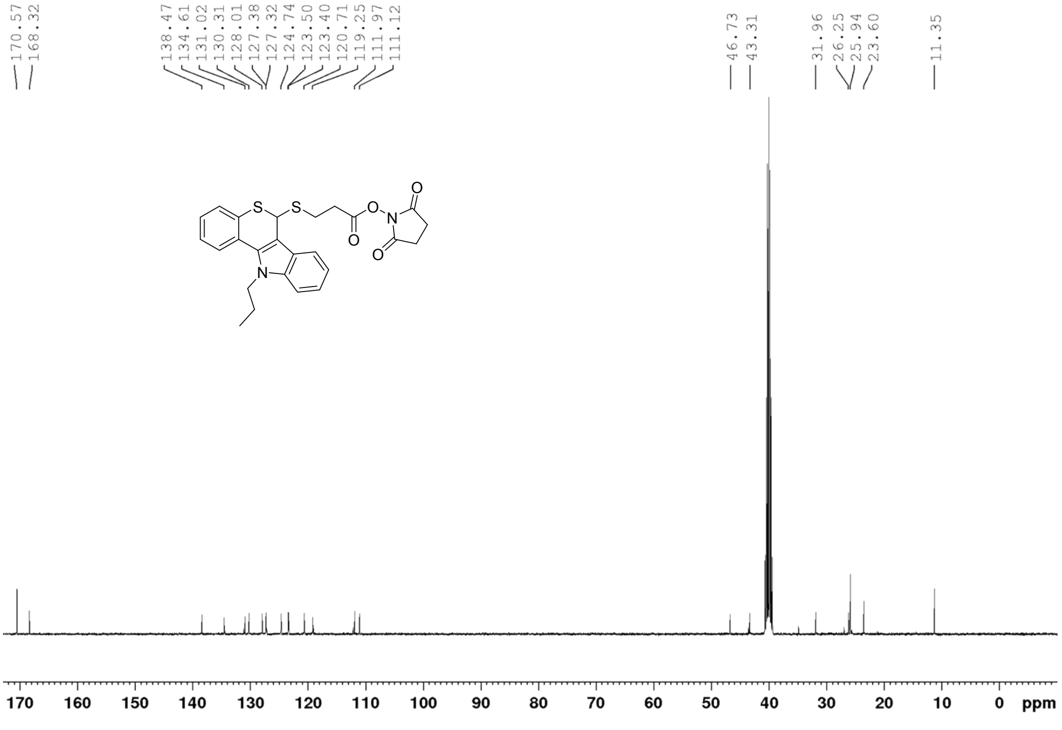

Figure S6. ^{13}C-NMR spectrum of probe **3** (100 MHz, 298 K, DMSO-d_6).

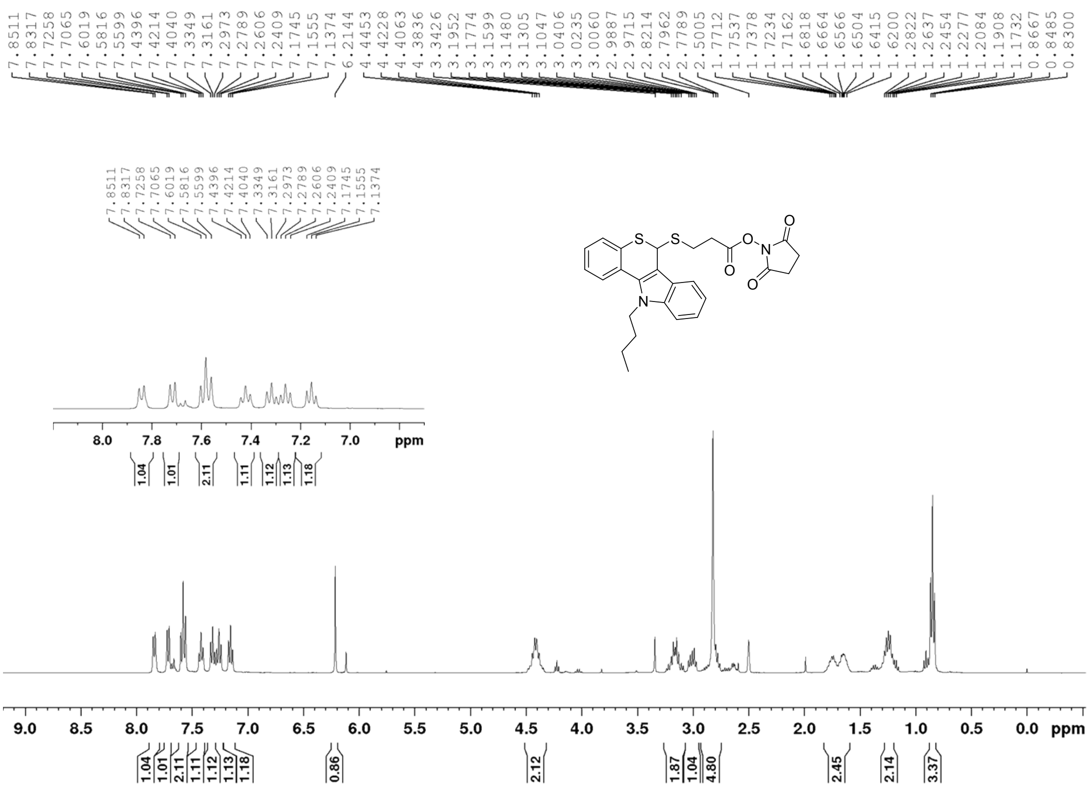

Figure S7. ^{1}H-NMR spectrum of probe **4** (400 MHz, 298 K, DMSO-d$_6$).

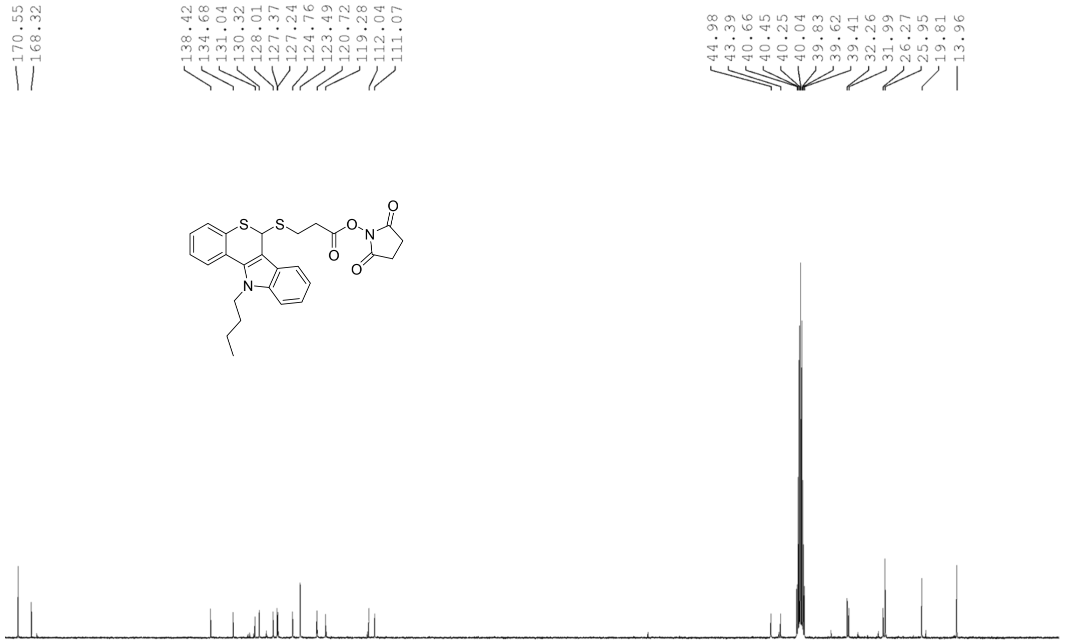

Figure S8. ^{13}C-NMR spectrum of probe **4** (100 MHz, 298 K, DMSO-d$_6$).

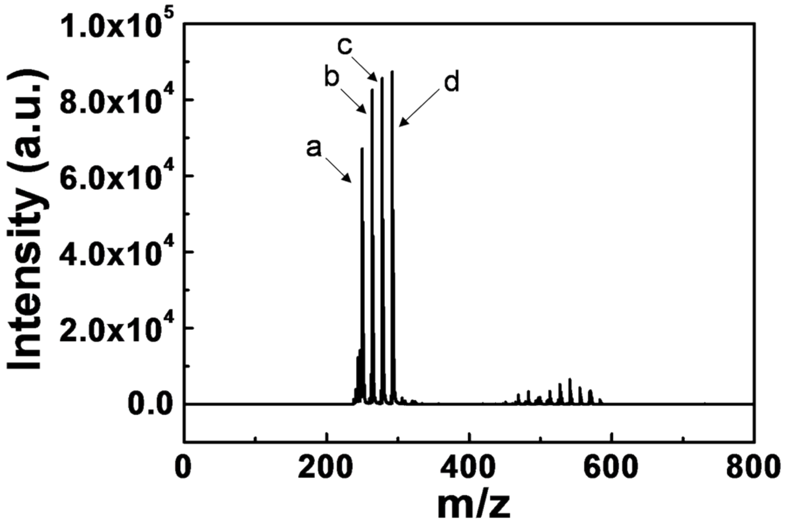

Figure S9. LDI-MS spectrum of mixture of an equal molar of probes **1-4.** a: probe **1**; b: probe **2**; c: probe **3**; d: probe **4**.

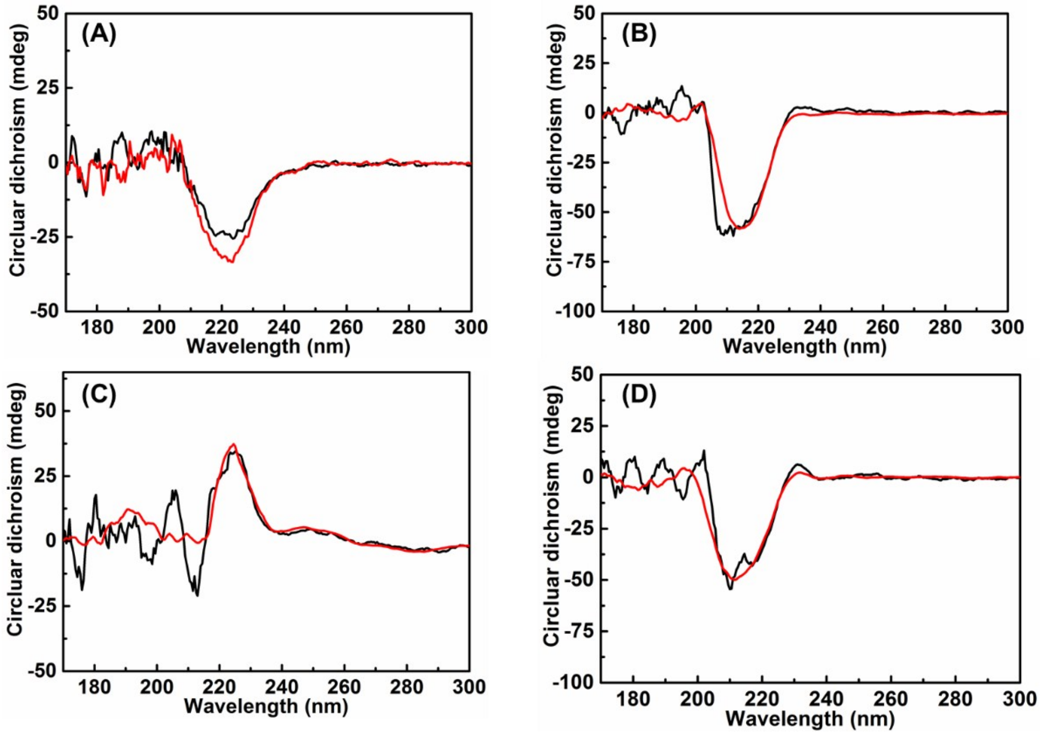

Figure S10. Circular dichroism of four lectins (red line), including concanavalin A (ConA), ricinus communis agglutinin (RCA_{120}), wheat germ agglutinin (WGA) and elderberry (SNA), and lectin-probes (black line), respectively .

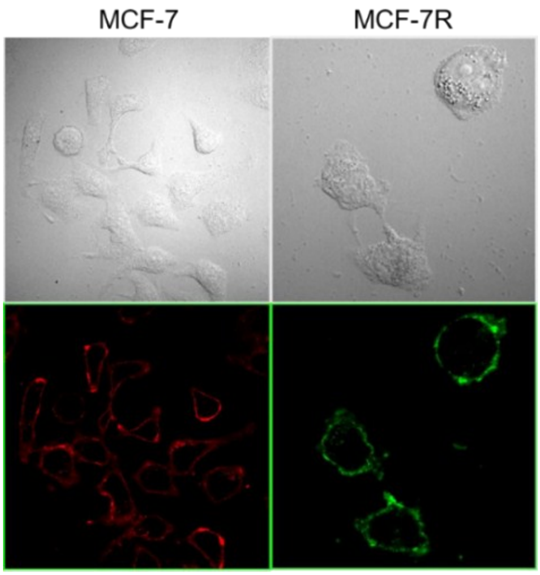

Figure S11. Confocal fluorescence images of MCF-7 and MCF-7R cells, which were incubated with FITC-labeled ConA-probe**1** for 40 min, respectively.

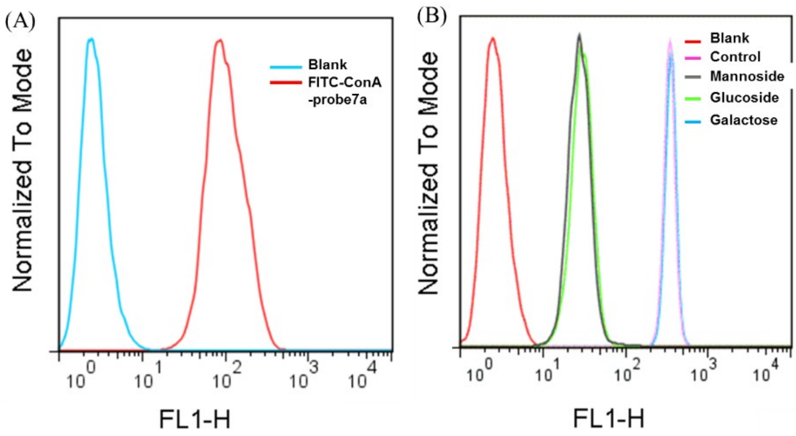

Figure S12. (A) Flow cytometry analysis shows the binding of FITC-labeled ConA-probe**1** (red) and untreated cells are shown in blue for comparison. (B) Monosaccharide inhibition assay. Flow cytometry analysis of FITC fluorescence in cells. Cells were incubated with FITC-labeled ConA-probe**1** pretreated by free monosaccharides (α-methyl-mannoside, α-methyl-glucoside and D-galactose).

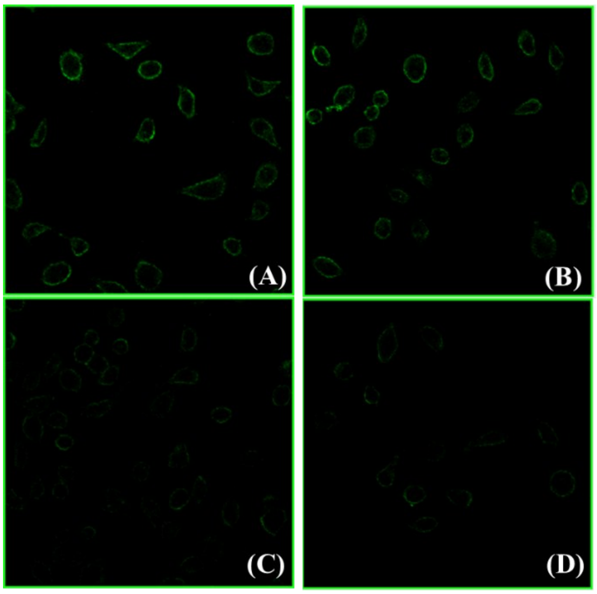

Figure S13. Confocal fluorescence images of MCF-7, which were incubated with FITC-labeled ConA-probe**1** pretreated by free monosaccharides (B) D-galactose, (C) α-methyl-mannoside, (D)α-methyl-glucoside and without pretreated (A).

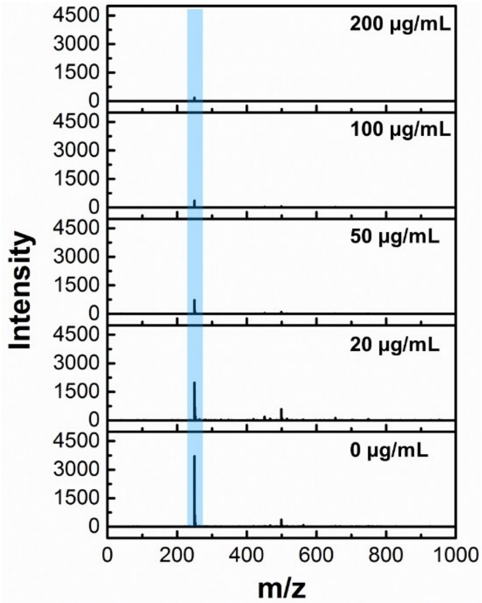

Figure S14. MS spectra of the amounts of α-mannosyl groups in cells under the stimuli of tunicamycin at different concentration.

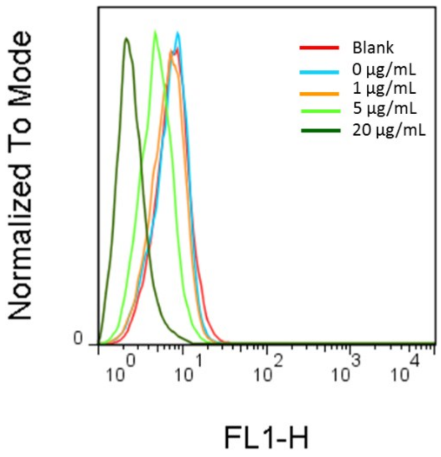

Figure S15. Flow cytometry analysis of expression of the α-mannosyl groups in cells under the stimuli of tunicamycin at different concentration.

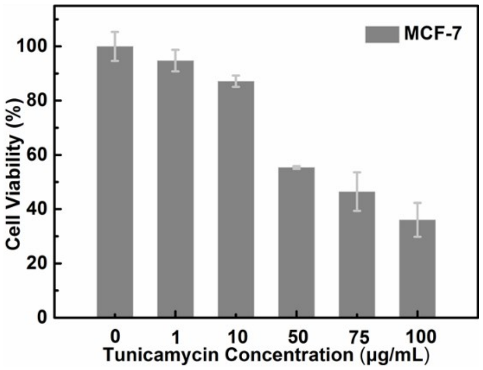

Figure S16. Effects of tunicamycin at varied concentrations on the viability of MCF-7 cells. The results are expressed as the mean ± SD (n = 5).

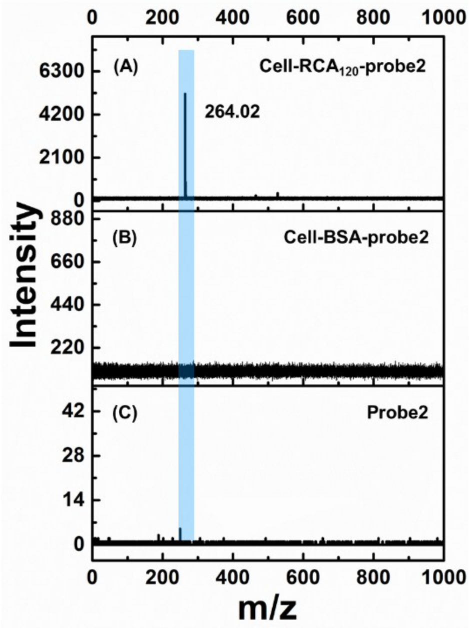

Figure S17. LDI-TOF MS analysis of the cell surface glycans based on laser cleavable probes. Mass spectrum of MCF-7 cells labeled by (A) RCA_{120}-probe**2**, (B) BSA-probe**2** and (C) probe**2**.

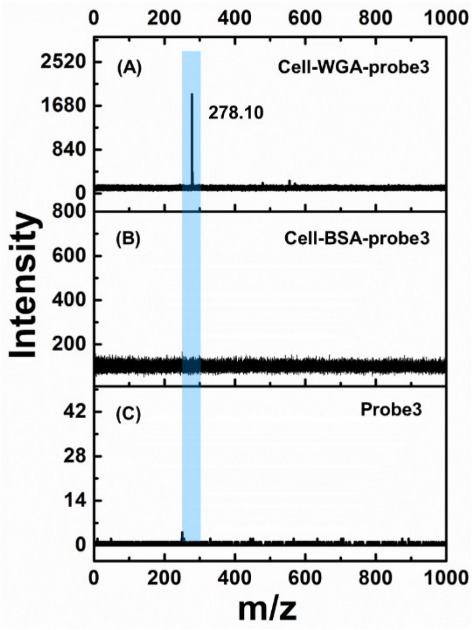

Figure S18. LDI-TOF MS analysis of the cell surface glycans based on laser cleavable probes. Mass spectrum of MCF-7 cells labeled by (A) WGA-probe**3**, (B) BSA-probe**3** and (C) probe**3**.

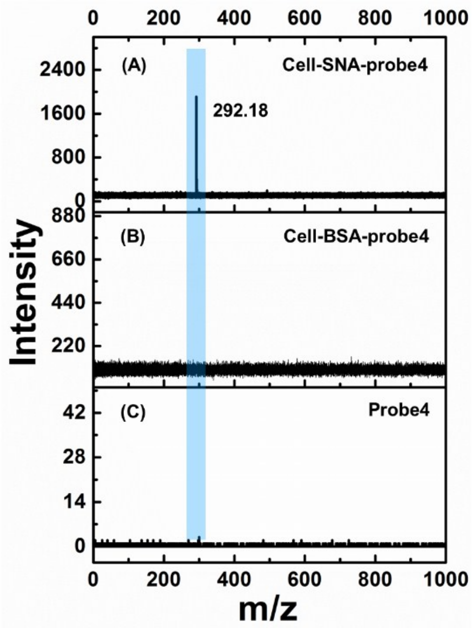

Figure S19. LDI-TOF MS analysis of the cell surface glycans based on laser cleavable probes. Mass spectrum of MCF-7 cells labeled by (A) SNA-probe**4**, (B) BSA-probe**4** and (C) probe**4**.

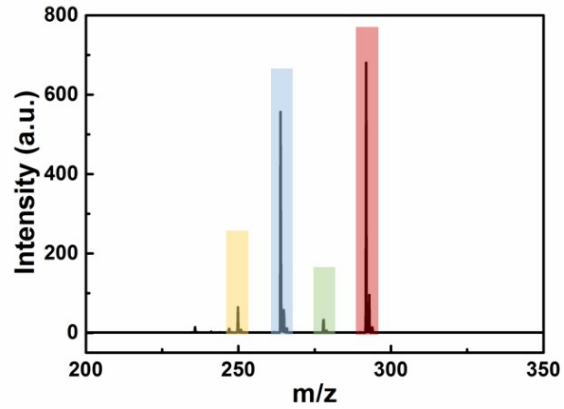

Figure S20. LDI-MS analysis of the cell surface glycans based on laser cleavable probes. Mass spectrum of MCF-7R cells labelled by the equal molar mixture of four lectin-probes.

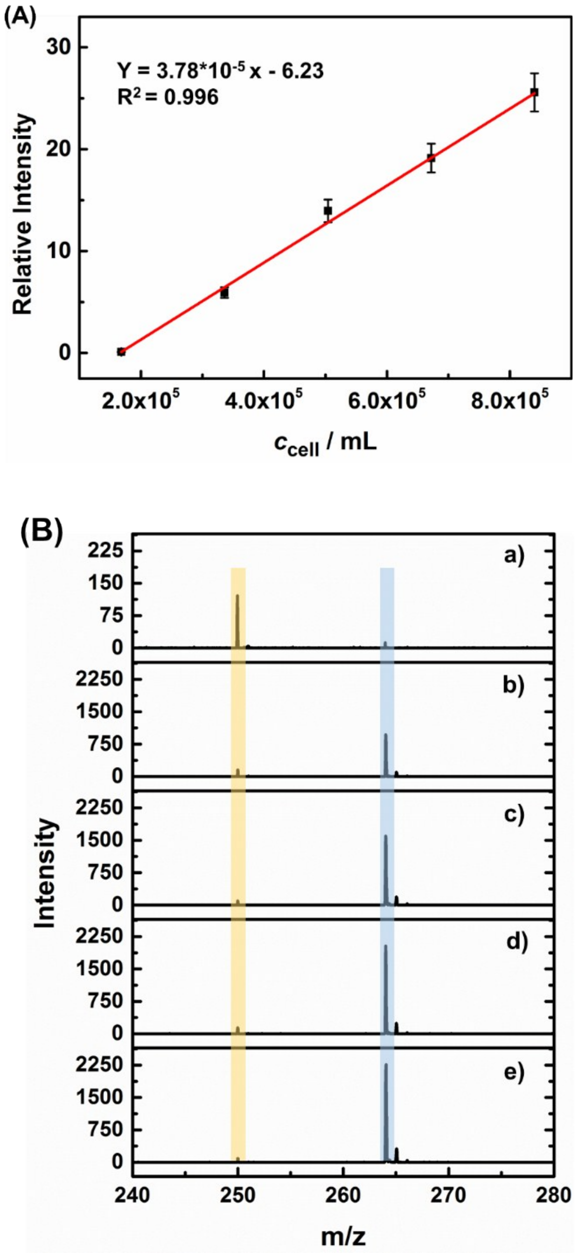

Figure S21. (A) Linear calibration curve of cell concentration. (B) LDI-MS analysis of cell precipitation at different concentration: a) 1.68E5 cells/mL, b) 3.36E5 cells/mL, c) 5.04E5 cells/mL, d) 6.72E5 cells/mL, e) 8.40E5 cells/mL. 100 μL cell suspensions were added in the experiment. Peak in orange color refers to internal standard ConA-probe**1**, and peak in blue refers to RCA_{120}-probe**2**.

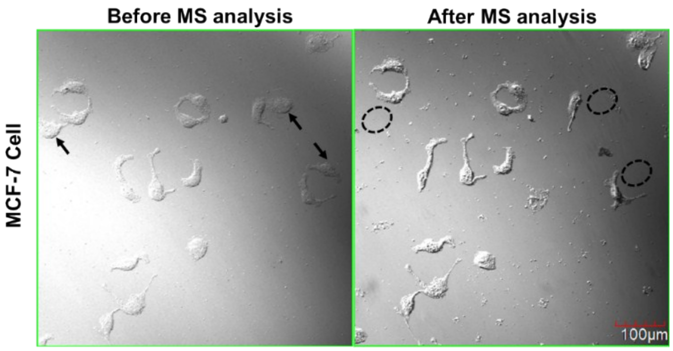

Figure S22. The optical imaging of MCF-7 cell before and after LDI-MS analysis. The arrows and circles indicate single cell disappeared during one shot laser by LDI-MS. Scale bar = 100 μm.

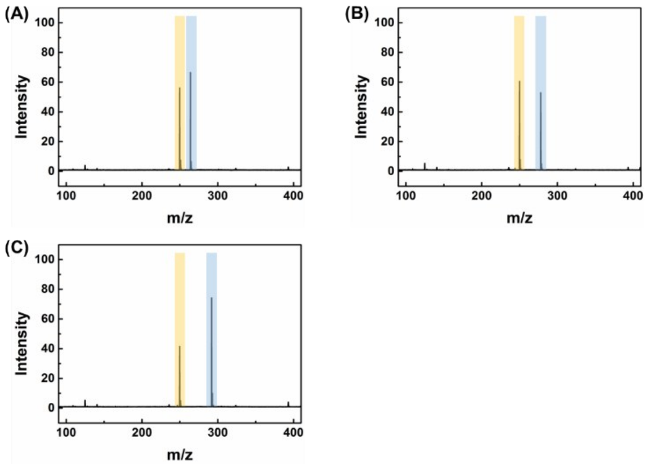

Figure S23. LDI-MS analysis of cell lysate with the equal molar mixture of (A) ConA-probe**1** and RCA_{120}-probe**2**, (B) ConA-probe**1** and WGA-probe**3** and (C) ConA-probe**1** and SNA-probe**4**.

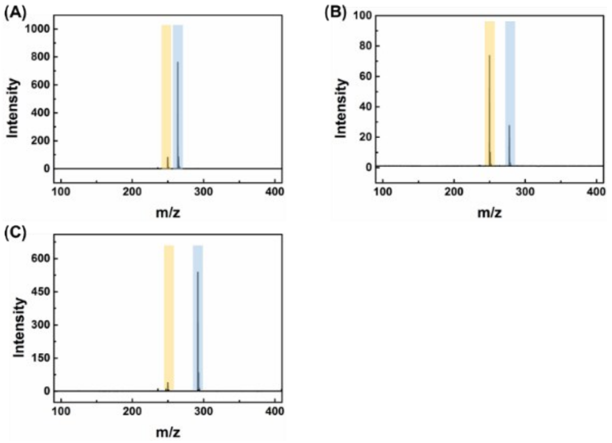

Figure S24. LDI-MS analysis of A) ConA-probe**1** and RCA_{120}-probe**2**, (B) ConA-probe**1** and WGA-probe**2** and (C) ConA-probe**1** and SNA-probe**4** on the surface of MCF-7 cell.

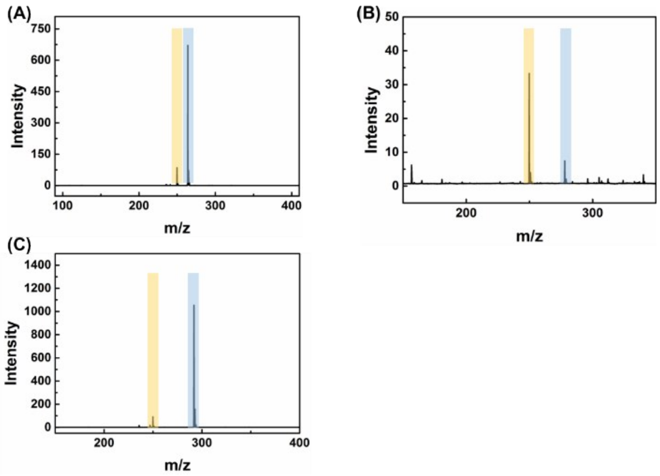

Figure S25. LDI-MS analysis of A) ConA-probe**1** and RCA_{120}-probe**2**, (B) ConA-probe**1** and WGA-probe**3** and (C) ConA-probe**1** and SNA-probe**4** on the surface of MCF-7R cell.

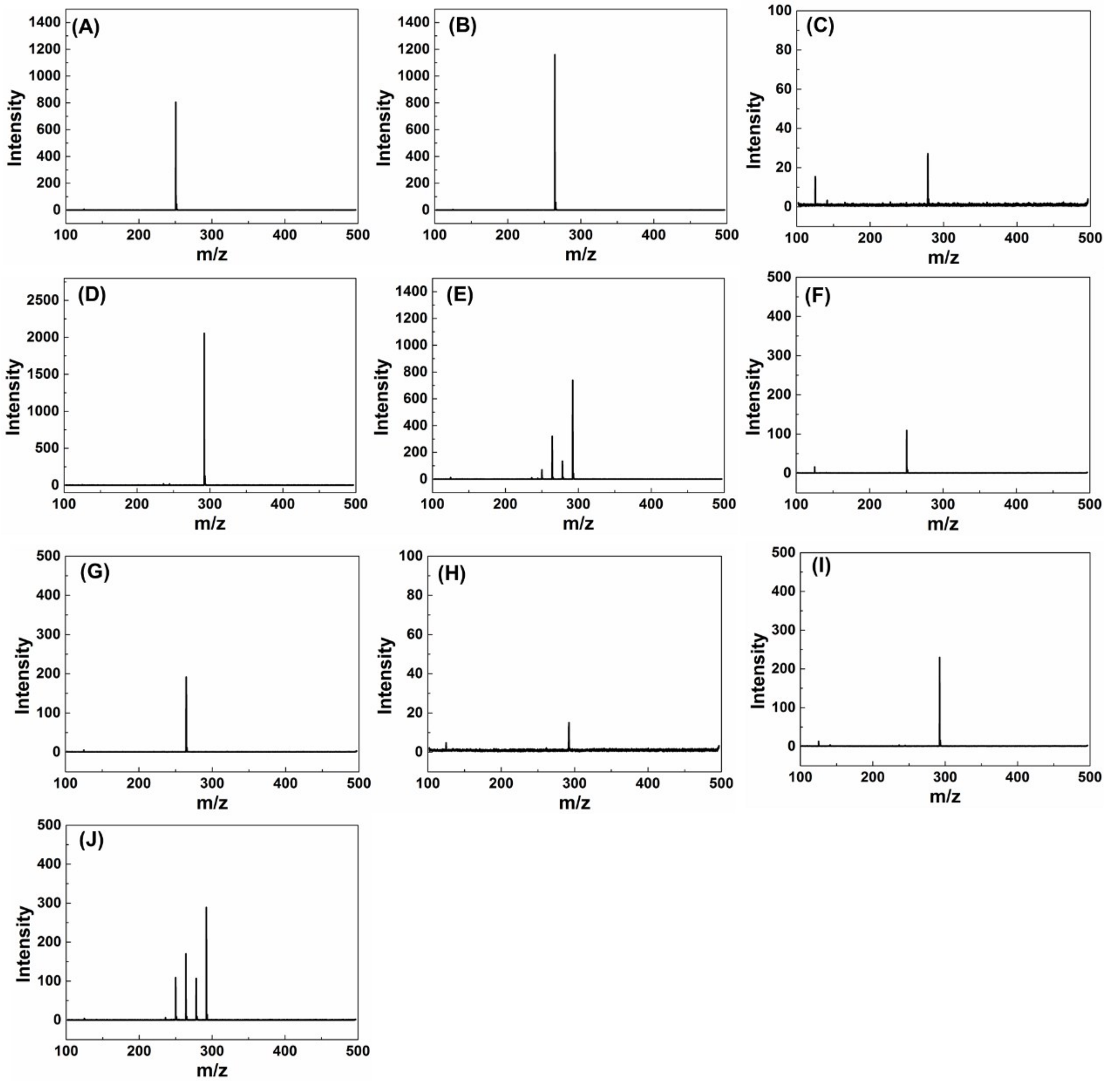

Figure S26. Average mass spectrum of the cancerous and paracancerous tissue, which were labeled by ConA-probe**1** (A and F), RCA_{120}-probe**2** (B and G), WGA-probe**3** (C and H), SNA-probe**4** (D and I) and an equal molar mixture of lectin-probes

(E and J).

Table S1. The diffusion coefficients of four probes and lectin-probes.

	Molecular weight/Da	Diffusion Coefficients /($\times 10^{-10}$ m^2/s)
Probe**1**	452.54	3.082
ConA-Probe**1**	≈102 k	2.316
Probe**2**	466.57	3.092
RCA-Probe**2**	≈120 k	2.748
Probe**3**	480.60	3.050
WGA-Probe**3**	≈36 k	2.574
Probe**4**	494.62	3.038
SNA-Probe**4**	≈140 k	2.747

Table S2. LDI-MS relative quantification of four types of glycans on the MCF-7 and MCF-7R cells by lectin-probe conjugates.

	RCA_{120}/ConA	WGA/ConA	SNA/ConA
control	1.28 ± 0.23	0.95 ± 0.25	2.03 ± 0.39
ER[a] of MCF-7	9.59 ± 2.34	0.36 ± 0.11	14.83 ± 4.10
RR[b] of MCF-7	7.49 ± 0.45	0.38 ± 0.02	7.31 ± 0.66
ER of MCF-7R	7.57 ± 2.01	0.47 ± 0.15	11.62 ± 3.59
RR of MCF-7R	5.91 ± 0.53	0.49 ± 0.03	5.72 ± 0.69

[a] ER is short for Experimental Ratio. [b] RR is short for Relative Ratio by correction.

Reference

1. N. Kang, J. M. Lee, A. Jeon, H. B. Oh, B. Moon, *Tetrahedron*, **2016**, *72*, 5612-5619.
